# Efficacy and safety of insulin degludec/aspart in patients with type 2 and type 1 diabetes mellitus: real-world evidence from Indonesia

**DOI:** 10.3389/fendo.2025.1690169

**Published:** 2025-11-10

**Authors:** Hendra Zufry, Krishna Wardhana Sucipto, Agustia Sukri Ekadamayanti, Qanita Iqbal

**Affiliations:** 1 Division of Endocrinology, Metabolism and Diabetes–Thyroid Center, Department of Internal Medicine, Faculty of Medicine, Universitas Syiah Kuala, Banda Aceh, Indonesia; 2 Division of Endocrinology, Metabolism and Diabetes–Thyroid Center, Department of Internal Medicine, Dr. Zainoel Abidin Hospital, Banda Aceh, Indonesia; 3 Innovation and Research Center of Endocrinology, Faculty of Medicine, Universitas Syiah Kuala, Banda Aceh, Indonesia

**Keywords:** diabetes, insulin degludec/aspart, IDegAsp, Indonesia, real-world data

## Abstract

**Background:**

Real-world studies on insulin degludec/aspart (IDegAsp) have been conducted in some Southeast Asian populations; however, data specific to Indonesia remain limited. The aim of this study was to evaluate the efficacy, safety profiles, and real-world clinical experience of IDegAsp after five years of implementation in diabetes care in Indonesia.

**Methods:**

This five-year, single-center, open-label, prospective, non-interventional study included adults with type 1 diabetes mellitus (T1DM) and type 2 diabetes mellitus (T2DM) who had been on IDegAsp treatment for at least 12 months. Glycemic and metabolic outcomes—glycated hemoglobin (HbA1c), fasting plasma glucose (FPG), postprandial glucose (PPG), and body mass index (BMI)—were assessed at baseline, 3, 6, and 12 months. The safety was evaluated based on hypoglycemia incidence. Clinical rationale for IDegAsp initiation and regimen models were also documented.

**Results:**

A total of 550 individuals (T1DM: 48; T2DM: 502) were included. At 12 months, both groups had significant reductions in HbA1c (T1DM: −3.60%, T2DM: −3.32%), FPG (T1DM: −119.39 mg/dL, T2DM: −105.60 mg/dL), and PPG (T1DM: −190.87 mg/dL, T2DM: −180.10 mg/dL) (all *p* < 0.001 compared to baseline). Slight but statistically significant increases in BMI were observed in both groups (both *p* < 0.001). No episodes of hypoglycemia were reported among T1DM patients, whereas in the T2DM cohort, it occurred in 3.0% of cases comprising 1.4% with a single episode and 1.6% with two episodes with no severe hypoglycemia reported. The most frequent reasons for initiating IDegAsp included suboptimal HbA1c and PPG levels, with T2DM patients more often citing the need for flexible injection time or schedule.

**Conclusion:**

IDegAsp demonstrated sustained glycemic improvement at 3-, 6-, and 12-months follow-ups with a favorable safety profile over one year, in both T1DM and T2DM populations in Indonesia. These findings support its utility in routine clinical practice, particularly among patients with unmet glycemic targets or complex treatment needs.

## 1 Introduction

Diabetes remains a major global health burden, with rising prevalence and substantial undiagnosed rates (1, 2). According to the International Diabetes Federation (IDF), 11.1% of adults aged 20–79 years—approximately 1 in 9—are living with diabetes in 2025, with over 40% remaining undiagnosed (3). Projections for 2050 estimate a substantial rise in prevalence, with 1 in 8 adults—or approximately 853 million people—expected to be living with diabetes, representing a 46% increase (3). In Indonesia, diabetes affected 11.3% of adults, equivalent to approximately 20.4 million individuals (4).

Insulin therapy remains a cornerstone in the management of both type 1 (T1DM) and type 2 diabetes mellitus (T2DM) (5). Despite the availability of various insulin regimens—including basal, bolus, and premixed formulations—clinical challenges persist, particularly in achieving glycemic targets while minimizing the risk of hypoglycemia (6–8). Existing regimens often require complex titration schedules, and patient adherence may be suboptimal due to dosing frequency or side effect profiles (7). In response to these limitations, dual-action insulin formulations have emerged as an alternative approach (9). Dual-action insulin modalities—including the co-formulation insulin degludec/aspart (IDegAsp)—have been introduced in many countries as a therapeutic option for both T1DM and T2DM (10–16). IDegAsp offers a simplified regimen with pharmacokinetic properties designed to address both fasting and postprandial hyperglycemia, which may enhance adherence and clinical outcomes (17). The increasing availability of IDegAsp in routine practice necessitates not only a comprehensive understanding of its pharmacologic profile but also the development of clinical skills in patient selection, dose titration, individualized management strategies and cost consideration (18). Optimal outcomes depend on the ability of healthcare providers to implement this modality effectively within the constraints of real-world settings.

To date, expert consensus, randomized controlled trials, multicenter studies, and post-marketing surveillance have provided a strong evidence supporting the efficacy and safety of IDegAsp (10–15, 18–40). However, the majority of these studies were conducted in high-resource settings under controlled clinical conditions, which may not adequately reflect the complexities and variability encountered in routine clinical practice. Although real-world studies exist in Southeast Asia (20, 21, 24, 33, 34), to the best of our knowledge, no study to date has specifically explored the Indonesian population. This represents a critical gap, given the distinct clinical, cultural, lifestyle, and systemic characteristics of diabetes care in Indonesia such as delayed insulin initiation, limited access to endocrinology specialists, and variable treatment adherence influenced by dietary patterns high in carbohydrates, low levels of physical activity, socioeconomic disparities, and health literacy challenges (41, 42). Long-term real-world data from Indonesia are essential to complement existing evidence, offering practical insights into the use of IDegAsp in a resource-limited healthcare system and guiding locally relevant clinical decision-making.

In Indonesia, IDegAsp has been fully covered under the national universal health coverage scheme since 2021, allowing broader access across diverse patient populations. Given the high and growing prevalence of diabetes in the country, insights into the long-term use of IDegAsp in clinical settings are especially relevant for informing policy, practice, and future research. Therefore, aim of this study was to evaluate the efficacy, safety profiles, and real-world clinical experience of IDegAsp after five years of use in diabetes care in Indonesia.

## 2 Materials and methods

### 2.1 Study design and setting

**Figure 1:**
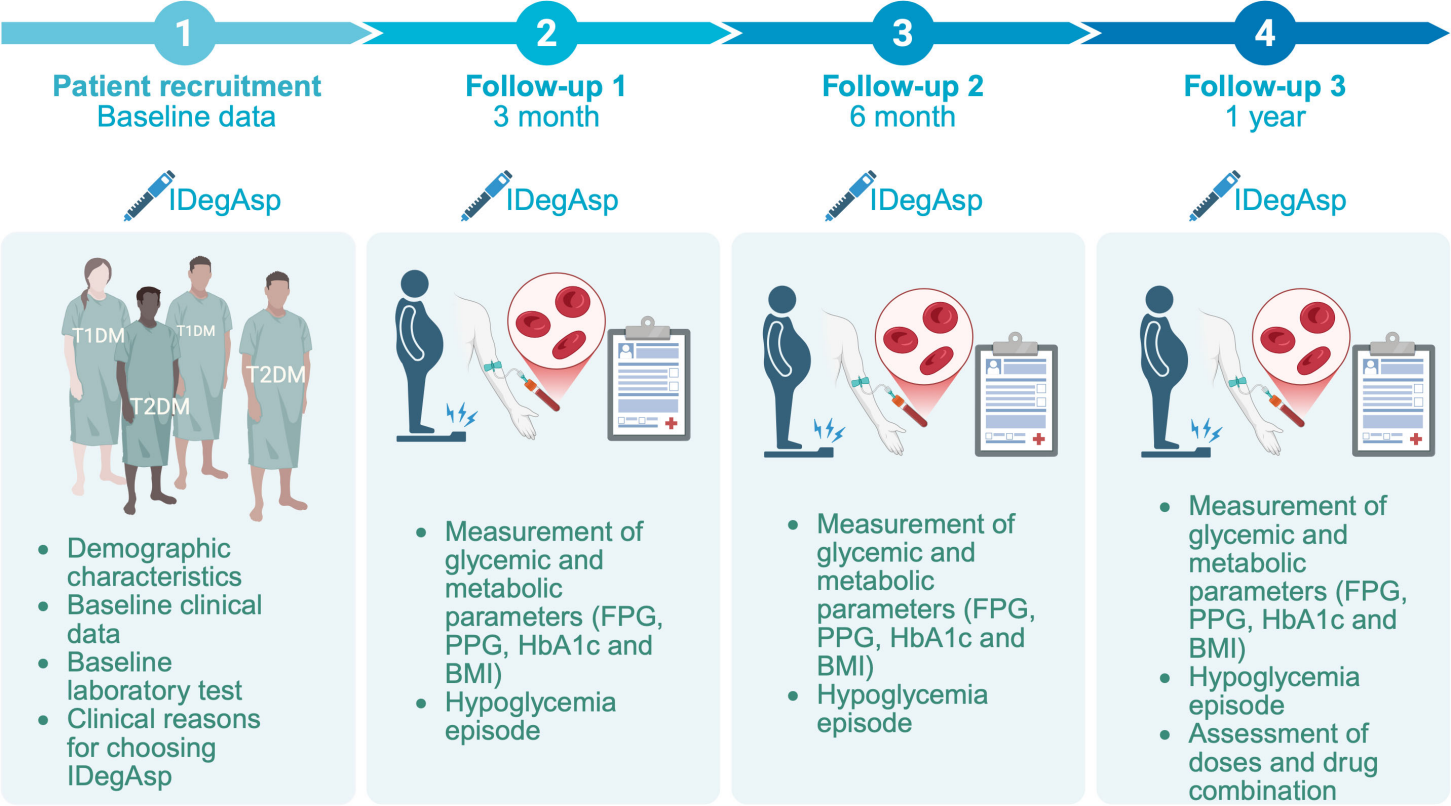
Study timeline and data collection points, including initiation of IDegAsp therapy and subsequent assessments of fasting plasma glucose (FPG), postprandial glucose (PPG), glycated hemoglobin (HbA1c), body mass index (BMI), hypoglycemia assessment in individuals with type 1 (T1DM) and type 2 diabetes mellitus (T2DM).

A five-years, single-center, open-label, prospective, non-interventional study was conducted. The objective was to evaluate the real-world efficacy and safety profile of IDegAsp in patients with T1DM and T2DM who had been receiving this insulin co-formulation continuously for at least 12 months (**Figure 1**). The study was conducted at the Outpatient Clinic of Endocrinology, Metabolism, and Diabetes, Dr. Zainoel Abidin Hospital, Banda Aceh, Indonesia, a tertiary and provincial referral hospital providing specialized diabetes care in Aceh, Indonesia. Data collection was performed over a five-year period, from January 2021 to May 2025. Clinical follow-up and data documentation covered a 12-month treatment duration for each enrolled patient. All clinical delivery were delivered as part of routine outpatient care by three board-certified internist-endocrinologists with varying lengths of professional experience: KWS (20 years), HZ (11 years), and ASED (3 years). No modifications were made to routine clinical care during the study period. Glycemic and metabolic outcomes, including fasting plasma glucose (FPG), postprandial glucose (PPG), and glycated hemoglobin (HbA1c) and body mass index (BMI), were assessed at baseline, 3, 6, and 12 months. The safety was evaluated based on hypoglycemia incidence.

### 2.2 Study size and participant criteria

Participants were recruited consecutively during routine outpatient visits according to predefined inclusion and exclusion criteria designed to reflect real-world clinical practice. Eligible participants were adults (≥18 years) with a diagnosis of T1DM or T2DM who were receiving antidiabetic medications other than IDegAsp. Additional inclusion criteria required an available HbA1c measurement obtained within 12 weeks prior to the baseline visit, defined as the time of informed consent and initiation of IDegAsp therapy. Exclusion criteria were prior treatment with IDegAsp, known hypersensitivity to the active substance or excipients listed in the local product label, and any condition that could impair understanding or cooperation, such as mental incapacity or language barriers. Patients could be withdrawn from the study due to withdrawal of informed consent or loss to follow-up.

### 2.3 Demographic and baseline clinical characteristics

Demographic and baseline clinical characteristics included sex, age, and duration of diabetes. Age was recorded in years and subsequently categorized into the following groups: 18–40, 41–50, 51–64, 65–74, and 75–84 years. Duration of diabetes was calculated in years from the time of initial diagnosis to the date of study enrollment. Renal function was evaluated using serum urea and creatinine levels (mg/dL), obtained through routine laboratory testing. Comorbidities such as stroke, diabetic retinopathy, Graves’ disease, coronary artery disease, pulmonary tuberculosis, hypertension, diabetic nephropathy, liver cirrhosis, diabetic neuropathy, and diabetic foot ulcer were documented. Diabetic neuropathy was assessed as part of routine clinical care, conducted using monofilament test (43).

### 2.4 Efficacy of insulin degludec/aspart

Glycemic and metabolic outcomes were assessed at baseline and at 3, 6, and 12 months, and included FPG, PPG, HbA1c and BMI. FPG and PPG were measured using venous plasma samples analyzed in a hospital-based clinical laboratory, and HbA1c was determined via high-performance liquid chromatography. BMI was calculated as weight in kilograms divided by height in meters squared (kg/m²), using measurements obtained during routine clinical visits.

### 2.5 Safety outcome: hypoglycemia incidence

Safety outcomes included the incidence of hypoglycemia. The incidence of hypoglycemia while using IDegAsp was evaluated for all patients. Hypoglycemia was defined as any documented event with plasma glucose at least <70 mg/dL. Any incidence of the severe hypoglycemia, plasma glucose <54 mg/dL, was also evaluated. These episodes were recorded through patient self-monitoring data and clinician documentation.

### 2.6 Indication for initiating insulin degludec/aspart

The decision to initiate or switch to IDegAsp was based on prior therapy, glycemic status, safety considerations, and convenience. In insulin-naive individuals on multiple oral antidiabetic agents, IDegAsp once daily was considered in cases of inadequate glycemic control—defined as FPG >130 mg/dL, PPG >180 mg/dL, or HbA1c >7.5%—especially when accompanied by symptoms such as polyuria, fatigue, or rising medication needs. IDegAsp was also used in cases of extreme hyperglycemia (glucose >300 mg/dL) or low BMI (<18.5 kg/m²).

For patients previously treated with basal insulin, premixed insulin, or basal-plus regimens, IDegAsp once daily was introduced to address persistent fasting or postprandial hyperglycemia and to minimize nocturnal hypoglycemia. Those on premixed insulin twice daily or basal-bolus therapy were shifted to intensified IDegAsp regimens to reduce glycemic variability and injection burden. In individuals with suboptimal postprandial control despite basal insulin, IDegAsp was added at the main meal. For those using glucagon-like peptide-1 (GLP-1) receptor agonists with poor glycemic control or intolerable side effects (e.g., nausea, vomiting, renal impairment), a switch to IDegAsp was considered.

Flexibility in injection timing was a factor in those with irregular schedules. A history of hypoglycemia (plasma glucose <70 mg/dL), older age, or renal impairment supported transition to IDegAsp. Treatment was also initiated due to dissatisfaction with prior regimens, including weight gain, edema, complexity, or poor practicality. Weight management was a consideration in patients with BMI ≥23.0 kg/m². Suspected beta-cell failure and prior adverse effects—such as recurrent hypoglycemia, gastrointestinal intolerance, or weight gain >2 kg within three months—were additional factors influencing the decision.

### 2.7 Distribution and total daily dose of insulin degludec/aspart regimens

Dosing patterns of IDegAsp were also assessed and classified according to frequency and the inclusion of additional aspart injection. Aspart refers to rapid-acting insulin used to manage postprandial glucose. In this study, dosing patterns of IDegAsp were classified by injection frequency and use of additional aspart for prandial control. The regimens included: one dose of IDegAsp alone, IDegAsp plus one or two doses of aspart, two doses of IDegAsp, or two doses of IDegAsp plus one dose of aspart.

### 2.8 Concomitant of antidiabetic drugs therapy

In addition, the patterns of concomitant antidiabetic therapy among T2DM patients were analyzed based on the number of oral antidiabetic drugs (OADs) used—none, monotherapy, dual therapy, or polytherapy. Documented OAD classes included metformin, sulfonylureas, thiazolidinediones (TZDs), acarbose, dipeptidyl peptidase-4 (DPP-4) inhibitors, and sodium-glucose co-transporter-2 (SGLT2) inhibitors. Although not classified as OADs, the use of GLP-1 receptor agonists was also recorded as part of the therapeutic profile.

### 2.9 Data collection

Clinical and laboratory data were collected both prospectively during routine outpatient visits. Collected variables included patient demographics, type and duration of diabetes, prior and current insulin regimens, BMI, HbA1c, FPG, PPG, total daily insulin dose (units/day), and comorbidities. Safety outcomes, including hypoglycemia (self-reported or confirmed episodes), were also documented. The study did not involve any intervention beyond standard clinical care. All treatment decisions, including insulin titration, follow-up intervals, and adjunctive therapies, were determined by the attending endocrinologist. Patients were evaluated monthly during routine clinic visits, and no protocol modifications were introduced during the study period. HbA1c and glucose parameters were measured using validated hospital laboratory methods. Insulin doses and hypoglycemia events were obtained from patient self-monitoring records and clinical documentation.

### 2.10 Statistical analysis

Continuous variables were presented as mean ± standard deviation or median with interquartile range, depending on the results of normality testing using the Kolmogorov-Smirnov test. Categorical variables were reported as frequencies and percentages. The primary outcomes were the change in glycemic and metabolic parameters over time: FPG, PPG, HbA1c and BMI. One-way ANOVA was applied across four time points (baseline, 3 months, 6 months, and 12 months), followed by *post hoc* analysis using Bonferroni correction to determine the time point with the most significant improvement and whether further improvement plateaued. To explore factors associated with hypoglycemia incidence, logistic regression analysis was performed of which plausible variables included were age, sex, baseline renal function, total daily insulin dose, and duration of diabetes. A *p* < 0.05 was considered statistically significant. All statistical analyses were performed using SPSS Statistics version 25.0 (IBM Corp., Armonk, NY, USA) and GraphPad Prism (GraphPad Software, San Diego, CA, USA) was used for data visualization.

## 3 Results

### 3.1 Characteristics of the patients

A total of 550 diabetic patients (48 of T1DM and 502 of T2DM) included in this study and their characteristics are presented in **Table 1**. The T1DM group had a higher proportion of males (62.5%), whereas in the T2DM group, the sex distribution was nearly equal (50.4% male and 49.6% female). The median age was 40 years (range: 22–66) in the T1DM group and 57 years (range: 31–81) in the T2DM group. More than half individuals with T1DM were aged 18–40 years (52.1%), while the majority of those with T2DM were in the 51–64 age group (63.3%). The median duration of diabetes was 8 years (range: 1–15) in T1DM and 10 years (range: 5–18) in T2DM. Median serum urea and creatinine levels were similar across both groups (urea: 39 mg/dL; creatinine: 1 mg/dL). Diabetic neuropathy was highly prevalent in both groups (97.9% in T1DM; 96.6% in T2DM). Coronary artery disease was the most common comorbidity in both T1DM (45.8%) and T2DM (53.0%), followed by hypertension (39.6% in T1DM; 43.8% in T2DM). Lung tuberculosis was more frequently observed in T1DM (29.2%) than in T2DM (7.0%). Other complications, including diabetic nephropathy, retinopathy, and diabetic ulcers, were also documented with varying prevalence in both groups (**Table 1**).

**Table 1:** Characteristics of the type 1 and type 2 diabetic patients included in this study (n=550).

Characteristics	Type 1 diabetes mellitus (n=48) Frequency (%)	Type 2 diabetes mellitus (n=502) Frequency (%)
Sex		
Male	30 (62.5)	253 (50.4)
Female	18 (37.5)	249 (49.5)
Age (years), median (min–max)	40 (22–66)	57 (31–81)
18–40	25 (52.1)	17 (3.4)
41–50	19 (39.6)	81 (16.1)
51–64	3 (6.3)	318 (63.3)
65–74	1 (2.1)	81 (16.1)
75–84	0 (0.0)	5 (1.0)
Disease duration (years), median (min-max)	8 (1–15)	10 (5–18)
Urea (mg/dL), median (min-max)	39 (32–64)	39 (31–90)
Creatinine (mg/dL), median (min-max)	1 (0.5–2.5)	1 (0.5–3.2)
Comorbidity		
Stroke	0 (0.0)	17 (3.4)
Diabetic retinopathy	1 (2.1)	58 (11.6)
Graves’ disease	0 (0.0)	4 (0.8)
Coronary artery disease	22 (45.8)	266 (53.0)
Lung tuberculosis	14 (29.2)	35 (7.0)
Hypertension	19 (39.6)	220 (43.8)
Diabetic nephropathy	5 (10.4)	77 (15.3)
Hepatic cirrhosis	3 (6.3)	10 (2.0)
Diabetic neuropathy	47 (97.9)	485 (96.6)
Diabetic ulcer	2 (4.2)	6 (1.2)

### 3.2 Efficacy of insulin degludec/aspart on glycemic and metabolic response improvement in individuals with type 1 and type 2 diabetes

**Figure 2:**
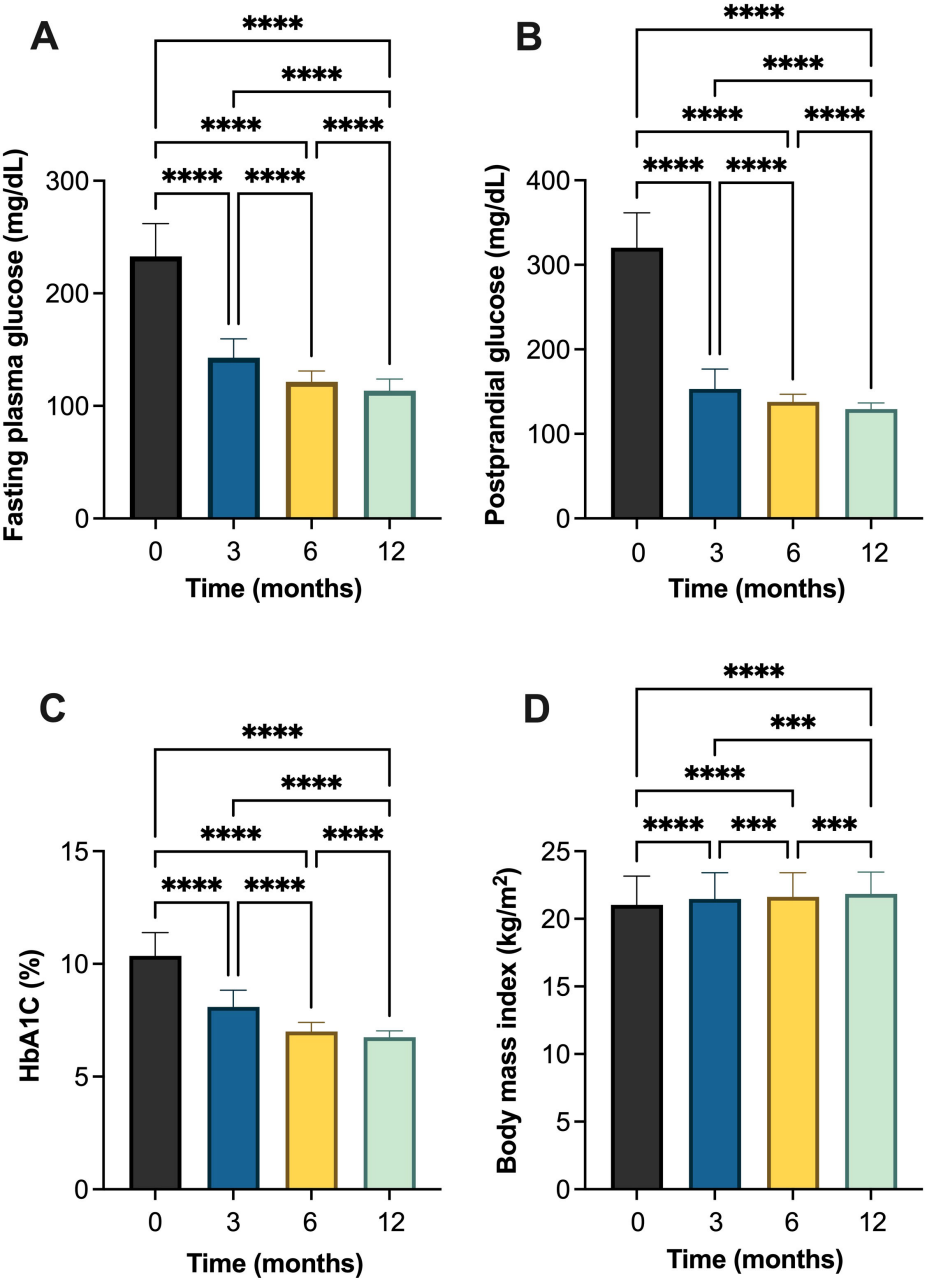
Changes in glycemic and metabolic parameters in individuals with type 1 diabetes mellitus (T1DM) following initiation of insulin degludec/aspart (IDegAsp): **(A)** fasting plasma glucose (FPG); **(B)** postprandial glucose (PPG); **(C)** glycated hemoglobin (HbA1c) (%); and **(D)** body mass index (BMI). *** Statistically significant at p<0.001; **** Statistically significant at p<0.0001.

Over a 12-month follow-up, there was a statistically significant improvement in all parameters in T1DM patients (**Table 2**, **Figure 2** and **Supplementary Table S1-S4**). FPG demonstrated a significant decline, from 232.89 ± 29.01 mg/dL at baseline to 113.50 ± 10.46 mg/dL after 12 months of follow-up, which also significantly different between follow-up times. Similarly, PPG decreased significantly from 320.22 ± 41.23 mg/dL to 129.35 ± 7.20 mg/dL. HbA1c also showed consistent and significant reductions, dropping from 10.35 ± 1.03% at baseline to 6.75 ± 0.27% at month 12. BMI increased slightly from 21.02 ± 2.13 kg/m² at baseline to 21.84 ± 1.60 kg/m² at the end of the study, with each interval showing a statistically significant difference (**Table 2**, **Figure 2**).

**Table 2:** Changes in glycemic and metabolic parameters in individuals with type 1 diabetes mellitus (T1DM) following initiation of insulin degludec/aspart (IDegAsp).

Variable	Mean ± SD				*p*-value^#^
	Baseline	Month 3	Month 6	Month 12	
Fasting plasma glucose (FPG) (mg/dL)	232.89 ± 29.01^a^	142.85 ± 16.74^b^	121.43 ± 9.61^c^	113.50 ± 10.46^d^	<0.001*
Postprandial glucose (PPG) (mg/dL)	320.22 ± 41.23^a^	153.10 ± 23.62^b^	137.97 ± 8.92^c^	129.35 ± 7.20^d^	<0.001*
HbA1c (%)	10.35 ± 1.03^a^	8.05 ± 0.70^b^	7.00 ± 0.40^c^	6.75 ± 0.27^d^	<0.001*
Body mass index (BMI) (kg/m^2^)	21.02 ± 2.13^a^	21.47 ± 1.93^b^	21.63 ± 1.77^c^	21.84 ± 1.60^d^	<0.001*

^#^Analyzed using one-way ANOVA.

*Statistically significant at *p* < 0.001.

^a-d^ Different letters indicate statistically significant differences between time points using *post hoc* Bonferroni correction analysis (*p* < 0.05).

**Figure 3:**
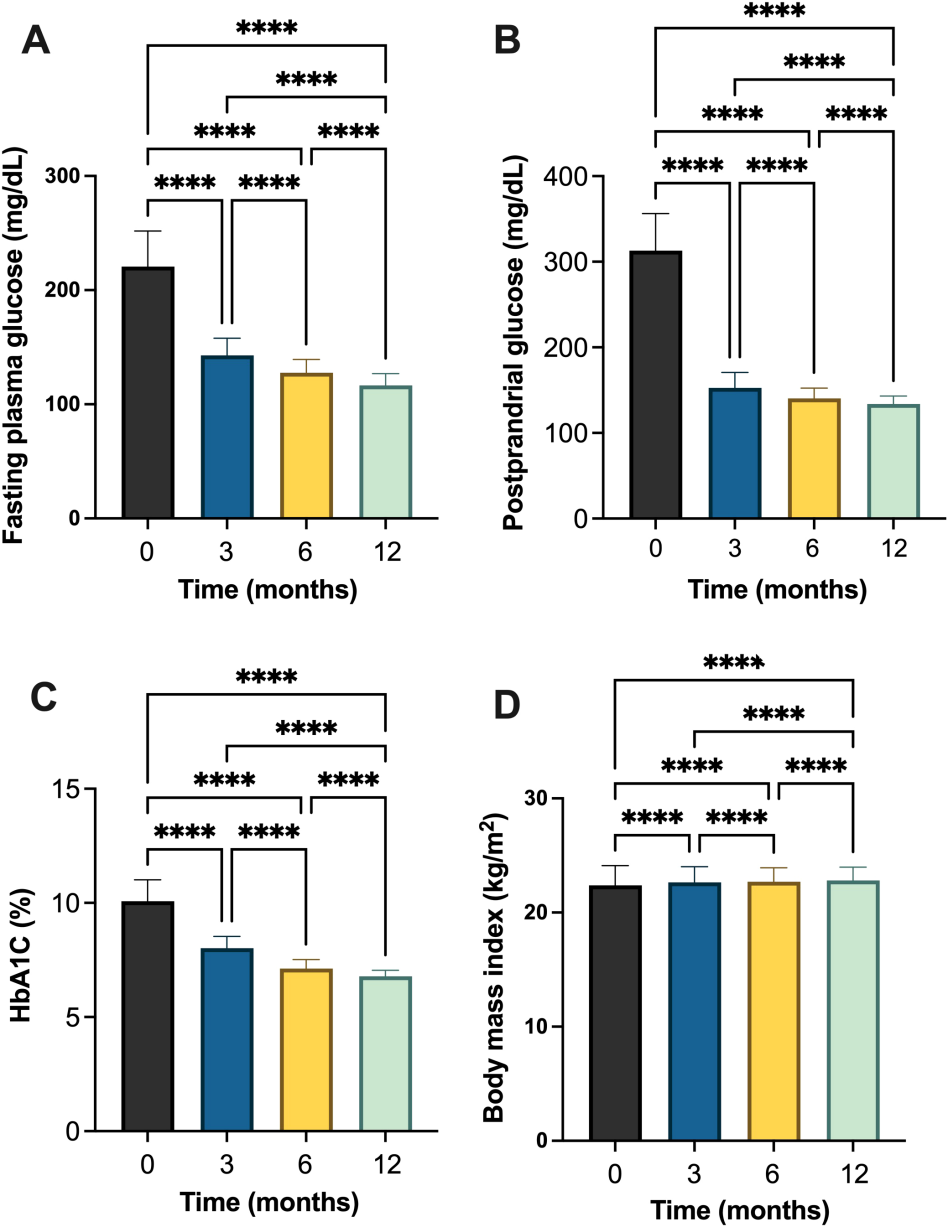
Changes in glycemic and metabolic parameters in individuals with type 2 diabetes mellitus (T2DM) following initiation of insulin degludec/aspart (IDegAsp): **(A)** fasting plasma glucose (FPG); **(B)** postprandial glucose (PPG); **(C)** glycated hemoglobin (HbA1c) (%); and **(D)** body mass index (BMI). **** Statistically significant at p<0.0001.

Similarly, in patients with T2DM, FPG levels decreased significantly from 221.80 ± 31.05 mg/dL at baseline to 116.2 ± 10.37 mg/dL at month 12, with statistically significant differences observed between consecutive timepoints indicating significant improvement in basal glycemic control (**Table 3**, **Figure 3** and **Supplementary Table S5-S8**). PPG levels also declined from 313.60 ± 43.13 mg/dL to 133.50 ± 9.05 mg/dL. HbA1c values showed a significant improvement trend, decreasing from 10.1 ± 0.94% at baseline to 6.78 ± 0.26% at the end of the study. BMI increased slightly from 22.27 ± 1.81 kg/m² at baseline to 22.72 ± 1.24 kg/m² at month 12, with statistically significant between timepoints (**Table 3**, **Figure 3**).

**Table 3:** Changes in glycemic and metabolic parameters in individuals with type 2 diabetes mellitus (T2DM) following initiation of insulin degludec/aspart (IDegAsp).

Variable	Mean ± SD				*p*-value^#^
	Baseline	Month 3	Month 6	Month 12	
Fasting plasma glucose (FPG) (mg/dL)	221.80 ± 31.05^a^	142.80 ± 15.33^b^	127.10 ± 11.64^c^	116.2 ± 10.37^d^	<0.001*
Postprandial glucose (PPG) (mg/dL)	313.60 ± 43.13^a^	152.6 ± 18.65^b^	140.10 ± 11.98^c^	133.50 ± 9.05^d^	<0.001*
HbA1c (%)	10.1 ± 0.94^a^	8.02 ± 0.53^b^	7.11 ± 0.39^c^	6.78 ± 0.26 ^d^	<0.001*
Body mass index (BMI) (kg/m^2^)	22.27 ± 1.81^a^	22.54 ± 1.46^b^	22.61 ± 1.30^c^	22.72 ± 1.24^d^	<0.001*

^#^Analyzed using one-way ANOVA.

*Statistically significant at *p* < 0.001.

^a-d^ Different letters indicate statistically significant differences between time points using *post hoc* Bonferroni correction analysis (*p* < 0.05).

### 3.3 Safety profile of insulin degludec/aspart in individuals with type 1 and type 2 diabetes mellitus

No episodes of hypoglycemia were reported among the all individuals with T1DM (**Table 4**). In contrast, among the 502 individuals with T2DM, 97.0% reported no hypoglycemia, while 1.4% experienced a single episode and 1.6% experienced two episodes (**Table 4**). No episodes were reported with glucose levels below 54 mg/dL (severe hypoglycemia), and no events required hospitalization. Age, duration of diabetes, serum creatinine levels, and total insulin dose were not significant predictors of hypoglycemia occurrence (*p*>0.05) (data not shown).

**Table 4:** Frequency of hypoglycemia episodes among individuals with type 1 (T1DM) and type 2 diabetes mellitus (T2DM) during the study period.

Hypoglycemia incidence	Type 1 diabetes mellitus (n=48) Frequency (%)	Type 2 diabetes mellitus (n=502) Frequency (%)
No hypoglycemia	0 (0)	487 (97)
Single episode	0 (0)	7 (1.4)
Two episodes	0 (0)	8 (1.6)

### 3.4 Clinical rationale for initiating insulin degludec/aspart in type 1 and type 2 diabetes mellitus

**Figure 4:**
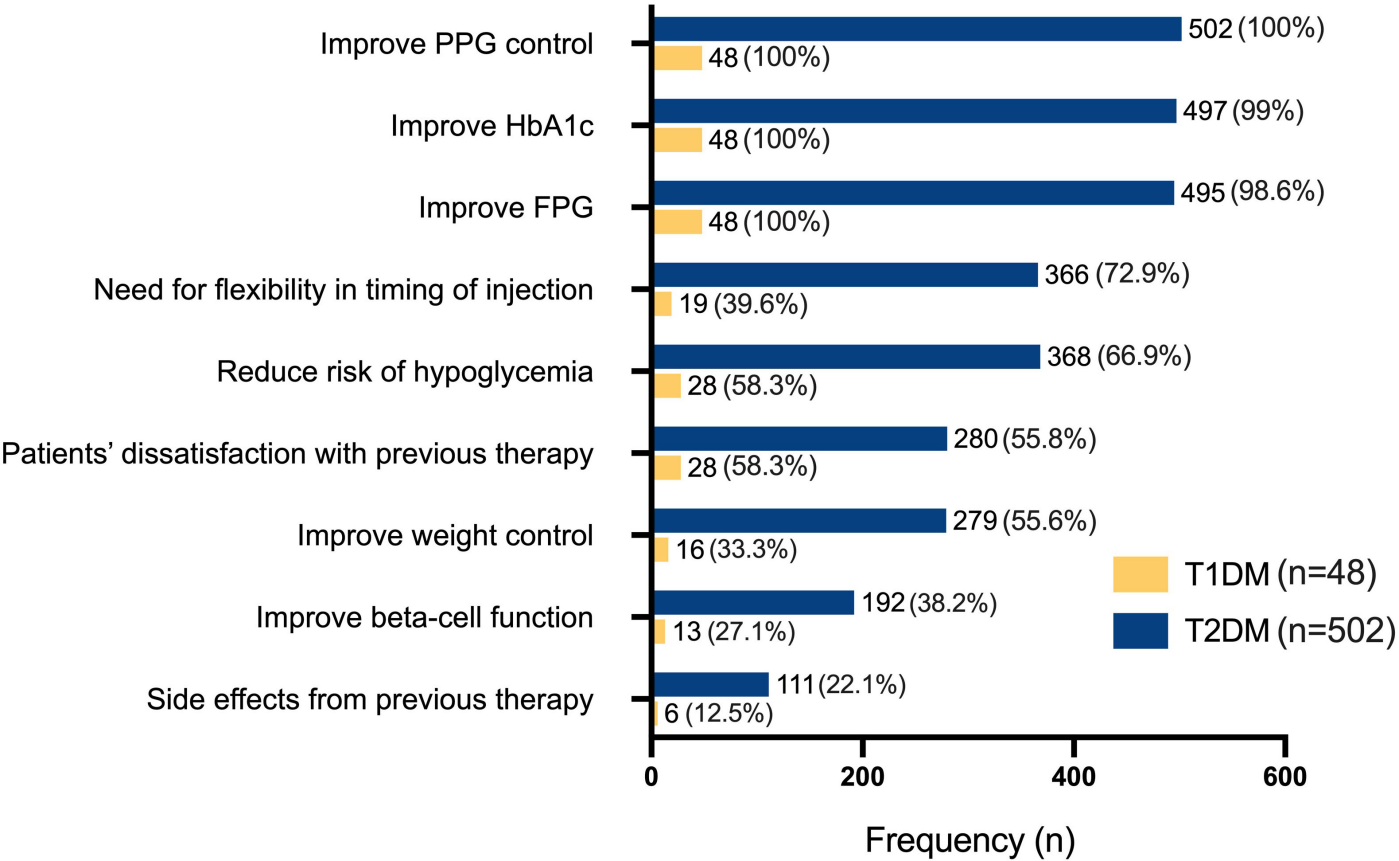
Clinical considerations for initiating insulin degludec/aspart (IDegAsp) therapy in individuals with type 1 (T1DM, n=48) and type 2 diabetes mellitus (T2DM, n=502). FPG, fasting plasma glucose; HbA1c, glycated hemoglobin; PPG, postprandial glucose.

In both T1DM and T1DM patients, the most commonly considered reasons were to improve PPG (100.0% in both) and HbA1c levels (99.0% in T2DM; 100.0% in T1DM), followed closely by the need to improve FPG (**Figure 4**). In T1DM, reducing hypoglycemia risk (58.3%) and dissatisfaction with previous therapy (58.3%) were the next most frequent reasons after glycemic targets. In T2DM, additional considerations included the need for flexible injection timing (72.9%), reduction of hypoglycemia risk (66.9%), and dissatisfaction with prior therapies (55.8%). Fewer individuals in either group considered weight control, beta-cell function improvement, or prior treatment-related side effects as reasons for initiating therapy.

### 3.5 Distribution and total daily dose of insulin degludec/aspart regimens in type 2 and type 1 diabetes mellitus

The most commonly used regimen in individuals with T1DM was 2 IDegAsp + 1 aspart, administered in 25 of 48 cases (52.1%), with a median total daily dose of 40 units (range: 28–60) (**Table 5**). Regimens requiring additional aspart injections, such as 1 IDegAsp + 2 aspart or 2 IDegAsp + 1 aspart, were more frequently used in T1DM (85.4%) than in T2DM (42.4%), reflecting the greater need for intensified prandial coverage in T1DM. In comparison, among 502 individuals with T2DM, the same 2 IDegAsp + 1 aspart regimen was also the most common (39.6%), with a higher median daily dose of 50 units (range: 28–60). Simpler regimen, such as 1 IDegAsp alone, was predominantly observed in T2DM (26.7%) and was infrequently used in T1DM (6.3%).

**Table 5:** Total daily dose of insulin degludec/aspart (IDegAsp) by regimen in individuals with type 1 (T1DM) and type 2 diabetes mellitus (T2DM).

Regimen	Total daily dose (unit/day)			
	Type 1 diabetes mellitus		Type 2 diabetes mellitus	
	Frequency	Median (min–max)	Frequency	Median (min–max)
1 IDegAsp	3	14 (14–14)	134	15.5 (12–34)
1 IDegAsp + 1 insulin aspart	1	–	5	18 (18–30)
1 IDegAsp + 2 insulin aspart	15	32 (18–34)	8	26 (18–34)
2 IDegAsp	4	51 (24–60)	156	40 (20–60)
2 IDegAsp + 1 insulin aspart	25	40 (28–60)	199	50 (28–60)

### 3.6 Concomitant of antidiabetic drugs therapy in type 2 diabetes mellitus

**Figure 5:**
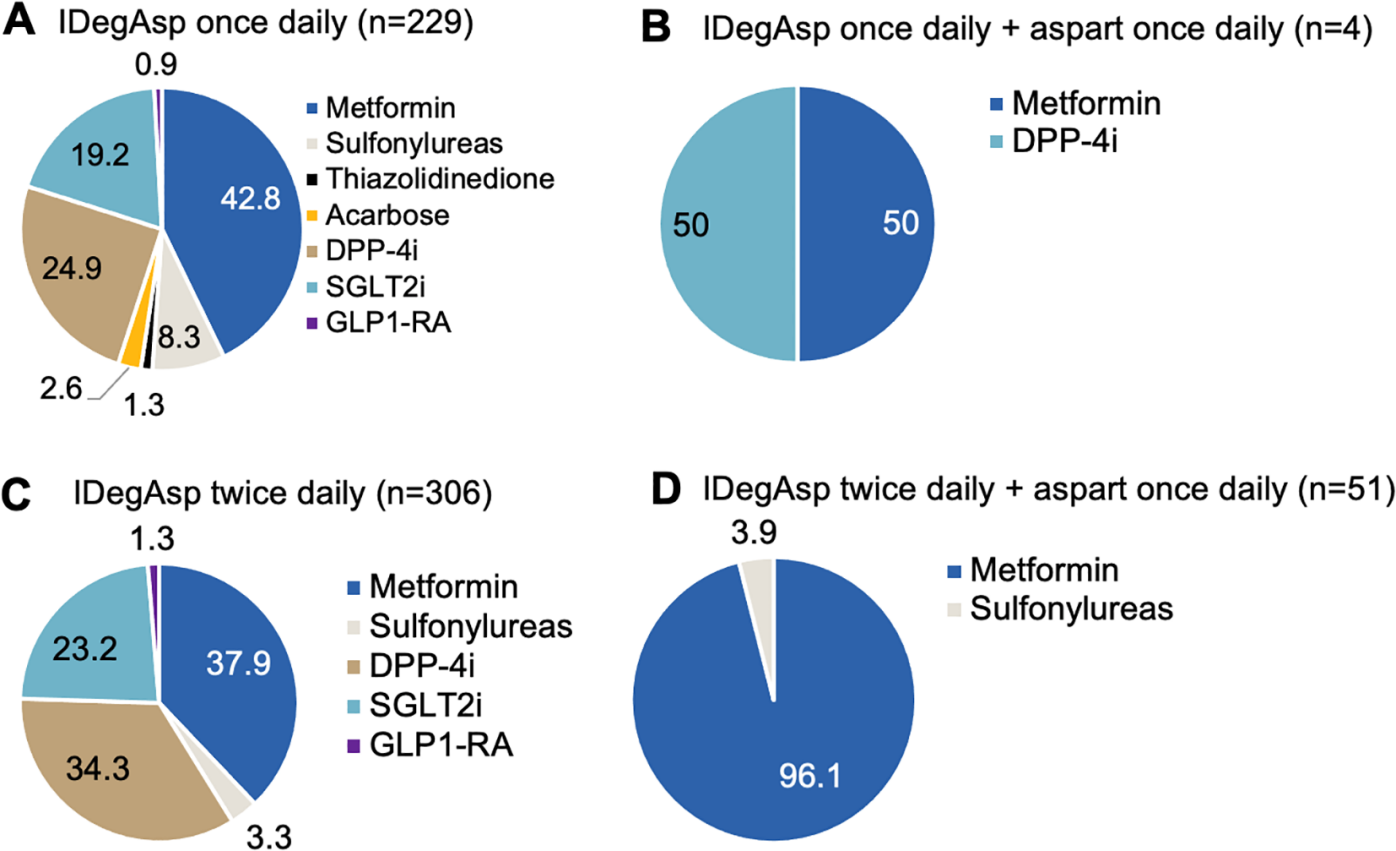
Concomitant oral antidiabetic drug (OAD) use in individuals with type 2 diabetes mellitus (T2DM) treated with insulin degludec/aspart (IDegAsp) once daily **(A, B)** or twice daily **(C, D)** with and without aspart insulin.

Next, OADs used concomitantly with various modifications of IDegAsp regimens among type 2 diabetic patients were recorded and the results are presented in **Figure 5**. Among individuals with T2DM receiving once-daily IDegAsp, concomitant use of OADs was common of which the most frequently used agents were metformin (42.8%), followed by DPP-4i (24.9%), SGLT2i (19.2%), and sulfonylureas (8.3%) (**Figure 5A**). Less frequent combinations included acarbose (2.6%), thiazolidinediones (1.3%), and GLP1-RA (0.9%). Among those treated with once-daily IDegAsp plus aspart, 50.0% received metformin and 50.0% received DPP-4i (**Figure 5B**). In individuals using twice-daily IDegAsp, 37.9% received metformin, 34.3% DPP-4i, 23.2% SGLT2i, 3.3% sulfonylureas, and 1.3% GLP1-RA (**Figure 5C**). Meanwhile, among those receiving twice-daily IDegAsp plus once-daily aspart, 96.1% were treated with metformin and 3.9% with sulfonylureas (**Figure 5D**).

### 3.7 Overall findings

**Figure 6:**
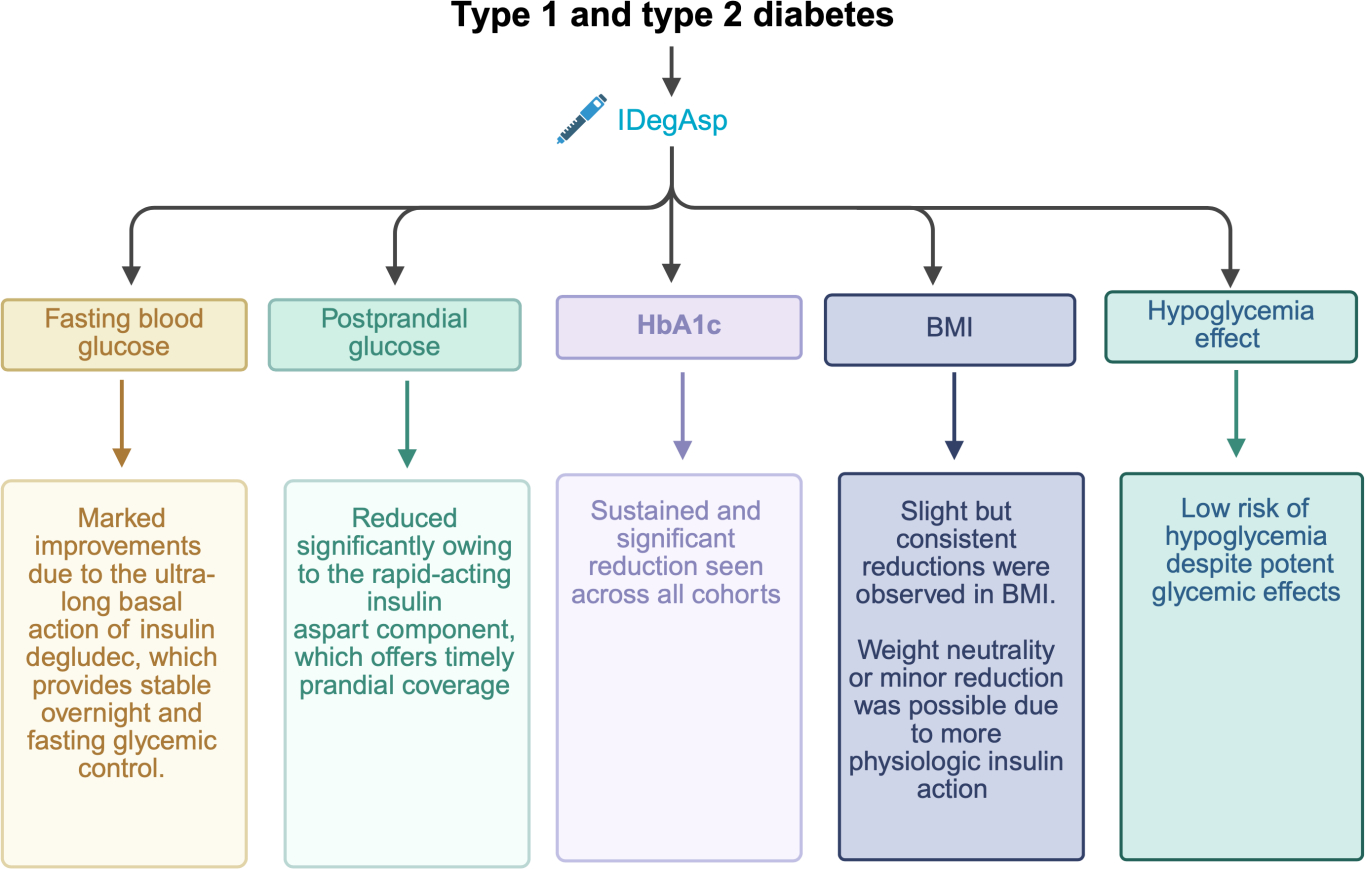
Summary of the real-world efficacy (glycemic and metabolic improvements) and safety of insulin degludec/aspart (IDegAsp) in Indonesian patients with type 1 (T1DM) and type 2 diabetes (T2DM).

The present real-world study assessed the clinical effectiveness and safety of IDegAsp in Indonesian individuals with diabetes. Over 12 months, both T1DM and T2DM groups showed significant improvements in FPG, PPG, HbA1c and BMI at months 3, 6, and 12. Glycemic control was consistently achieved in T1DM and T2DM, with progressive reductions across all timepoints, supporting the sustained effectiveness of IDegAsp in this population. The summary of efficacy and safety of IDegAsp from our study are presented in **Figure 6**.

## 4 Discussion

The pharmacodynamic and pharmacokinetic properties of IDegAsp play a central role in its glycemic benefits. IDegAsp combines the ultra-long-acting basal insulin degludec with the rapid-acting prandial insulin aspart in a soluble co-formulation (10). This profile allows for a stable and predictable basal glucose-lowering effect, while providing timely prandial coverage (10). These attributes contribute to sustained reductions in FPG, PPG and HbA1c observed in both T1DM and T2DM. In T2DM, characterized primarily by insulin resistance and progressive beta-cell dysfunction, the basal component of IDegAsp addresses fasting hyperglycemia by suppressing endogenous glucose production, while the prandial component helps to mitigate postprandial excursions (23). In contrast, T1DM is defined by near-complete beta-cell failure, necessitating full basal and prandial insulin replacement (44).

Most previous studies involving IDegAsp have predominantly focused on individuals with T2DM, consistently demonstrating substantial improvements in glycemic parameters (11, 12, 15, 20, 24–26, 30, 33, 34, 36, 37, 39, 40, 45). In particular, Asian populations have shown favorable responses, likely due to pathophysiological characteristics such as lower insulin resistance and earlier beta-cell dysfunction (12, 46–48). In Korea, switching to IDegAsp resulted in significant improvements in HbA1c and FPG among patients with T2DM duration of 18.9 years, with the proportion of individuals achieving HbA1c <7% increasing from 5.10% to 11.22% (*p* = 0.012), without significant weight gain or an increase in hypoglycemia (11). Similar glycemic benefits were observed in a Japanese study, where HbA1c reduction reached −0.51% after one year (*p* < 0.0001), particularly in individuals aged <75 years, those with renal impairment, those transitioning from premixed or basal-only regimens (12).

Similar outcomes were observed in multiple other regional cohorts. In China, a large cohort demonstrated a mean HbA1c reduction of −1.27% (*p* < 0.0001), with the greatest improvement observed in insulin-naïve individuals previously managed with OADs (−2.01%; *p* < 0.0001) (26). Indian data similarly showed a significant HbA1c decline of −1.6% (*p* < 0.0001) (20). Southeast Asian populations also had comparable benefits. In Malaysia, HbA1c was reduced by −1.3% (95%CI: −1.61 to −0.90; *p* < 0.0001), accompanied by a significant FPG decrease of −1.8 mmol/L (*p* < 0.0001), while the proportion of individuals achieving HbA1c <7% increased from 5.5% to 17.0% (33). In the Philippines, HbA1c decreased significantly by −1.4% (95%CI: −1.7 to −1.1; *p* < 0.0001), and FPG dropped by −46.1 mg/dL (*p* < 0.0001) (34). The mean duration of diabetes among participants was 10.8 ± 7.3 years in the Philippines (34), 11.2 ± 7.9 years in Malaysia (33), and 14.4 ± 8.1 years in India (20), indicating had long-standing T2DM at baseline.

In contrast, outcomes in individuals with T1DM remain inconclusive. A Japanese study failed to show a significant improvement in HbA1c (9.3 ± 1.7% to 9.6 ± 1.9%, *p*>0.05) or BMI, suggesting limited benefit (12). However, the present study adds valuable evidence supporting the efficacy of IDegAsp in T1DM, as significant reductions in FPG, PPG and HbA1c were observed over 12 months. These discrepancies across studies may be attributed to differences in baseline glycemic control, insulin dosing strategies, and the limited prandial flexibility of co-formulations in T1DM management.

In the present study, both T1DM and T2DM cohorts had notably higher baseline HbA1c and FPG levels than those reported in most real-world studies from other Asian countries. For instance, baseline HbA1c values in Indian, Malaysian, and Philippine cohorts ranged from 8.6% to 10.1%, with mean reductions of −1.3% to −1.6% after 6–12 months of IDegAsp therapy (20, 33, 34). Similarly, the Japanese long-term study reported a mean HbA1c reduction of −0.51% (12). The larger decrease observed in our population (−3%) is likely attributable to higher baseline hyperglycemia and delayed insulin initiation in Indonesia, where insulin is often started late in the disease course (41, 42). Additionally, as this was a tertiary-referral setting, patients typically presented with uncontrolled diabetes after multiple prior treatment failures. This setting also explains the very high prevalence of diabetic neuropathy in our cohort, which was screened using the monofilament test. The marked HbA1c improvement may further reflect intensive insulin titration practices in our center, where endocrinologists directly adjusted IDegAsp doses with close patient monitoring and communication every 2–3 days during the early treatment phase. Such proactive follow-up allowed timely dose optimization and facilitated rapid attainment of glycemic targets while maintaining a very low incidence of hypoglycemia. This phenomenon may also represent, in part, a regression-to-mean tendency, whereby individuals with markedly elevated baseline values experience proportionally greater absolute reductions following therapeutic intensification. Collectively, these factors likely contributed to the magnitude of HbA1c reduction observed in this real-world cohort.

Baseline glycemic control, insulin dosing strategies, and the degree of residual beta-cell function vary across study cohorts. T1DM is characterized by near-total pancreatic beta-cell loss, requiring precise and individualized prandial insulin adjustments (44). This need may not be fully met by the co-formulation of IDegAsp, which provides a basal-to-prandial insulin ratio that may lack sufficient flexibility, particularly in individuals with fluctuating carbohydrate intake or high prandial insulin requirements. Moreover, the relatively lower proportion of insulin aspart in the co- formulation may lead to suboptimal postprandial glucose regulation compared to conventional basal–bolus regimens. In studies where IDegAsp was used once or twice daily, total daily insulin dose adjustments were limited by concerns of hypoglycemia or rigidity in titration, which may also contribute to the lack of efficacy in certain T1DM populations (12, 14).

Several factors may have contributed to the variability in glycemic outcomes observed in the present study. Differences in baseline glycemic control, such as initial FPG, PPG and HbA1c levels, likely influenced the magnitude of improvement achieved following IDegAsp initiation. The type of diabetes also played a role, as T1DM and T2DM present distinct pathophysiological profiles—absolute insulin deficiency versus insulin resistance—which necessitated different dosing intensities and regimen structures (44, 46–48). Additionally, prior insulin exposure varied across participants, with some switching from premixed, basal-only, or basal–bolus therapies, potentially affecting the degree of responsiveness to IDegAsp. Variability in dosing frequency, particularly between once-daily and twice-daily regimens, further influenced glycemic outcomes, with twice daily dosing generally associated with greater HbA1c reductions.

Other contributing factors included the duration of diabetes, which may associate with residual beta-cell function and influence insulin needs. Adherence to insulin administration and self-monitoring practices, inherent to real-world settings, likely introduced further heterogeneity in treatment effects (20, 26). Titration strategies, which were physician-guided and subject to clinical judgment, patient preference, and local practices, also may played a role in outcome variability. Moreover, the presence of comorbidities and the use of concomitant oral antidiabetic agents may have modified treatment responses.

Another relevant consideration is the effect of IDegAsp on body weight. In the present study, a slight but statistically significant increase in BMI was observed over 12 months although all of them still within normal BMI. This finding may be attributed to the insulin aspart component of IDegAsp, which has been associated with increased appetite. Additionally, weight gain could reflect an early manifestation of improved glycemic control. Nevertheless, this finding contrasts with several previous real-world studies, which reported either no significant change or modest weight reduction—particularly among OAD-only users—the potential for weight gain remains a clinical concern when prandial insulin is introduced (11, 33, 34). Therefore, careful selection of concomitant oral agents, favoring weight-neutral or weight-lowering agents such as SGLT2 or DPP-4 inhibitors, remains important. Combination therapy strategies were tailored to minimize metabolic burden, a critical factor in optimizing long-term adherence and safety.

Furthermore, OAD use patterns among T2DM patients indicated that metformin was most common, followed by DPP-4i and SGLT2i across all regimens. These OAD combinations may have contributed to variations in glycemic control, insulin dosing, and weight changes in the present study.

In the present study, among 502 individuals with T2DM, 97.0% reported no hypoglycemic events, while 1.4% and 1.6% experienced one and two episodes, respectively; none involved glucose <54 mg/dL or required hospitalization. No hypoglycemia was reported among the 48 individuals with T1DM, highlighting the favorable safety profile of IDegAsp. These findings are consistent with previously published real-world evidence that demonstrated the hypoglycemia-sparing properties of IDegAsp, with an overall hypoglycemia incidence of 2.4% (12). The reported event rates were 7.6 per 100 patient-years of exposure in T1DM and 3.5 per 100 patient-years of exposure in T2DM (12). Compared to premixed or basal insulins, IDegAsp has consistently shown equivalent or superior HbA1c reduction, while significantly lowering the incidence of nocturnal hypoglycemia (49). This advantage is attributable to its distinct pharmacokinetic and pharmacodynamic profile, which combines 70% insulin degludec (ultra–long-acting) with 30% insulin aspart (rapid-acting) (15, 22). The formulation provides more physiologic postprandial coverage and reduces glycemic variability (21). Importantly, it also minimizes the so-called “shoulder effect,” a pharmacodynamic phenomenon observed with premixed insulins in which overlap between intermediate-acting and rapid-acting components leads to a prolonged insulin peak and increases the risk of postprandial hypoglycemia. The aspart component of IDegAsp ensures rapid and predictable prandial coverage immediately after injection, while the degludec component delivers a stable basal effect with minimal variability. This balanced action lowers the risk of hypoglycemia, which is particularly relevant in older adults with type 2 diabetes, where fear of hypoglycemia often hinders timely insulin intensification (10). In the present study, conducted in a population consuming predominantly carbohydrate-rich, rice-based meals, the 70:30 ratio of basal to prandial insulin in IDegAsp was generally sufficient to achieve glycemic control—especially for the main meal—with additional prandial coverage supported as required through oral antidiabetic drugs or supplementary aspart injections.

In T1DM, in the present study, where prandial control is particularly challenging due to absolute insulin deficiency, reducing the risk of hypoglycemia (58.3%) and dissatisfaction with prior therapies (58.3%) were key considerations. These responses reflect clinical challenges in T1DM management, where the balance between effective glycemic control and hypoglycemia prevention remains difficult to achieve, especially with rigid or less physiological insulin regimens (7). For T2DM, additional motivating factors included the flexibility in injection timing offered by IDegAsp (72.9%), which is beneficial in real-world settings where rigid schedules are often impractical. The preference for this flexibility suggests that treatment adherence and quality of life are important aspects influencing insulin regimen selection. The reduction of hypoglycemia risk (66.9%) and dissatisfaction with prior therapies (55.8%) were also prominent factors, indicating that despite relatively preserved endogenous insulin production in T2DM, safety and convenience remain key drivers in treatment decisions. Interestingly, fewer individuals from either group selected secondary factors such as weight control, beta-cell function preservation, or adverse effects from previous treatment as primary reasons for switching. This may be due to a greater emphasis on immediate glycemic targets and treatment burden rather than long-term pathophysiologic modulation.

In the present study, T1DM patients required more complex IDegAsp-based regimens with lower insulin doses, reflecting the need for intensified prandial control. In contrast, T2DM patients more often used simpler regimens with higher total daily doses, aligning with differing pathophysiological demands. Several real-world studies, particularly among insulin-experienced patients transitioning from basal-bolus or premixed regimens, have reported modest reductions in total, basal, or prandial insulin doses after switching to IDegAsp, though not all findings reached statistical significance (12, 24–26, 33, 39, 40). These discrepancies likely reflect heterogeneity in prior treatment exposure, titration protocols, and clinical decision-making across regions.

The primary distinction between real-world studies and randomized controlled trials lies in treatment adherence and patient selection. In randomized controlled trials, compliance tends to be higher due to structured protocols, close monitoring, and frequent follow-up visits. Participants are usually selected based on strict inclusion and exclusion criteria, often excluding individuals with multiple comorbidities, poor adherence, or complex treatment needs. In contrast, real-world settings reflect broader and more heterogeneous patient populations, where adherence may be influenced by external factors such as cost, access to care, lifestyle, and health literacy. Despite these challenges, the present study demonstrated sustained glycemic improvements and a low incidence of hypoglycemia over the long term, suggesting that IDegAsp remains clinically effective even in routine practice. These findings highlight the practical advantages of IDegAsp in real-world conditions, where treatment intensification is frequently limited by concerns about hypoglycemia risk, injection burden, and variable adherence.

The current findings support the positioning of IDegAsp as a preferred insulin option in clinical scenarios requiring both basal and prandial coverage without the complexity of full basal–bolus regimens. The flexibility of IDegAsp in dosing schedules, reduced need for injection frequency, and its suitability for patients with suboptimal adherence or fear of injections may enhance therapeutic satisfaction and long-term persistence. Previous studies have shown that treatment satisfaction scores improve after switching to IDegAsp, with improved glycemic control cited as the primary driver.

While these results are encouraging, certain limitations must be acknowledged. First, this was a single-center investigation conducted in a tertiary referral hospital in Aceh, which may limit the generalizability of the results to broader populations and diverse healthcare settings across Indonesia. Multicenter studies are therefore needed to validate these observations. Second, the single-arm, non-interventional design restricts the ability to establish direct comparisons with other insulin regimens and reduces the strength of causal inferences. Nonetheless, this approach was chosen to reflect real-world practice in Indonesia, where patient management is highly heterogeneous. Third, although concomitant OAD use was documented, longitudinal data on discontinuation, dose adjustments, and prior insulin regimens after initiation of IDegAsp were not systematically captured, precluding a complete evaluation of treatment optimization strategies. Fourth, no hypoglycemic episodes were reported among individuals with T1DM, a finding that may reflect under-reporting in routine practice, as mild or asymptomatic episodes are often unrecognized or insufficiently documented. Fifth, subgroup comparisons between the different IDegAsp regimens in T2DM (once daily, twice daily, or twice daily plus aspart) were not undertaken, as regimen choice was influenced by baseline glycemic levels and clinical characteristics, which would have introduced significant confounding. Finally, the study cohorts included both insulin-naïve and insulin-experienced individuals, which may have affected the magnitude of glycemic improvement observed. Future research should incorporate subgroup analyses to disentangle these effects and provide a clearer assessment of IDegAsp efficacy without confounding from prior insulin exposure.

In conclusion, five years of experience using IDegAsp demonstrated optimal efficacy and a low incidence of hypoglycemia when applied appropriately—beginning with proper patient selection and followed by effective titration using simple methods. The diverse utilization patterns of IDegAsp, within treatment regimens covered by Indonesia’s Universal Health Coverage system, support its suitability for broader implementation in many developing countries. Although the use of newer antidiabetic agents such as SGLT2 inhibitors and GLP-1 receptor agonists was limited in this study, the findings suggest that IDegAsp remains a practical and effective option in resource-limited settings.

## Data Availability

The raw data supporting the conclusions of this article will be made available by the authors, without undue reservation.
