# Supplementary material for: Efficacy and safety of insulin degludec/aspart in patients with type 2 and type 1 diabetes mellitus: real-world evidence from Indonesia
Source: Front Endocrinol (Lausanne). 2025 Nov 10;16:1690169. doi: 10.3389/fendo.2025.1690169 (PMC12640829; doi:10.3389/fendo.2025.1690169)
Supplement: Supplementary file 1 [file Table1.pdf]

Table S1. Post hoc analysis of Body mass index (BMI) among DMT1 patients

		Mean Difference	SE	P value.[b]	95% CI for Difference[b]	
					Lower Bound	Upper Bound
Baseline	Month 3	-.450*	.078	<.001	-.665	-.235
	Month 6	-.608*	.096	<.001	-.873	-.344
	Month 12	-.823*	.119	<.001	-1.150	-.496
Month 3	Baseline	.450*	.078	<.001	.235	.665
	Month 6	-.158*	.038	<.001	-.264	-.052
	Month 12	-.373*	.079	<.001	-.592	-.154
Month 6	Baseline	.608*	.096	<.001	.344	.873
	Month 3	.158*	.038	<.001	.052	.264
	Month 12	-.215*	.047	<.001	-.345	-.085
Month 12	Baseline	.823*	.119	<.001	.496	1.150
	Month 3	.373*	.079	<.001	.154	.592
	Month 6	.215*	.047	<.001	.085	.345

Based on estimated marginal means
*. The mean difference is significant at the .05 level.
b. Adjustment for multiple comparisons: Bonferroni.

Table S2. Post hoc analysis of fasting blood glucose (FBG) among DMT1 patients

		Mean Difference	SE	P value.[b]	95% CI for Difference[b]	
					Lower Bound	Upper Bound
Baseline	Month 3	90.042*	4.255	<.001	78.322	101.761
	Month 6	111.458*	4.545	<.001	98.939	123.977
	Month 12	119.396*	4.516	<.001	106.957	131.834
Month 3	Baseline	-90.042*	4.255	<.001	-101.761	-78.322
	Month 6	21.417*	2.162	<.001	15.462	27.371
	Month 12	29.354*	2.186	<.001	23.333	35.376
Month 6	Baseline	-111.458*	4.545	<.001	-123.977	-98.939
	Month 3	-21.417*	2.162	<.001	-27.371	-15.462
	Month 12	7.938*	1.060	<.001	5.019	10.856
Month 12	Baseline	-119.396*	4.516	<.001	-131.834	-106.957
	Month 3	-29.354*	2.186	<.001	-35.376	-23.333
	Month 6	-7.938*	1.060	<.001	-10.856	-5.019

Based on estimated marginal means
*. The mean difference is significant at the .05 level.
b. Adjustment for multiple comparisons: Bonferroni.

Table S3. Post hoc analysis of postprandial blood glucose (PBG) among DMT1 patients

		Mean Difference	SE	P value.[b]	95% CI for Difference[b]	
					Lower Bound	Upper Bound
Baseline	Month 3	167.125*	5.462	<.001	152.080	182.170
	Month 6	182.250*	5.499	<.001	167.102	197.398
	Month 12	190.875*	6.121	<.001	174.015	207.735
Month 3	Baseline	-167.125*	5.462	<.001	-182.170	-152.080
	Month 6	15.125*	2.949	<.001	7.002	23.248
	Month 12	23.750*	3.646	<.001	13.708	33.792
Month 6	Baseline	-182.250*	5.499	<.001	-197.398	-167.102
	Month 3	-15.125*	2.949	<.001	-23.248	-7.002
	Month 12	8.625*	1.144	<.001	5.473	11.777
Month 12	Baseline	-190.875*	6.121	<.001	-207.735	-174.015
	Month 3	-23.750*	3.646	<.001	-33.792	-13.708
	Month 6	-8.625*	1.144	<.001	-11.777	-5.473

Based on estimated marginal means
*. The mean difference is significant at the .05 level.
b. Adjustment for multiple comparisons: Bonferroni.

Table S4. Post hoc analysis of HbA1C among DMT1 patients

		Mean Difference	SE	P value.[b]	95% CI for Difference[b]	
					Lower Bound	Upper Bound
Baseline	Month 3	2.263*	.111	<.001	1.955	2.570
	Month 6	3.348*	.146	<.001	2.946	3.749
	Month 12	3.600*	.147	<.001	3.195	4.005
Month 3	Baseline	-2.263*	.111	<.001	-2.570	-1.955
	Month 6	1.085*	.088	<.001	.843	1.328
	Month 12	1.337*	.101	<.001	1.060	1.615
Month 6	Baseline	-3.348*	.146	<.001	-3.749	-2.946
	Month 3	-1.085*	.088	<.001	-1.328	-.843
	Month 12	.252*	.036	<.001	.153	.351
Month 12	Baseline	-3.600*	.147	<.001	-4.005	-3.195
	Month 3	-1.337*	.101	<.001	-1.615	-1.060
	Month 6	-.252*	.036	<.001	-.351	-.153

Based on estimated marginal means
*. The mean difference is significant at the .05 level.
b. Adjustment for multiple comparisons: Bonferroni.

Table S5. Post hoc analysis of body mass index (BMI) among DMT2 patients

		Mean Difference	SE	P value.[b]	95% CI for Difference[b]	
					Lower Bound	Upper Bound
Baseline	Month 3	-.256*	.029	<.001	-.333	-.178
	Month 6	-.323*	.037	<.001	-.420	-.225
	Month 12	-.422*	.046	<.001	-.543	-.300
Month 3	Baseline	.256*	.029	<.001	.178	.333
	Month 6	-.067*	.012	<.001	-.100	-.034
	Month 12	-.166*	.028	<.001	-.241	-.092
Month 6	Baseline	.323*	.037	<.001	.225	.420
	Month 3	.067*	.012	<.001	.034	.100
	Month 12	-.099*	.023	<.001	-.159	-.039
Month 12	Baseline	.422*	.046	<.001	.300	.543
	Month 3	.166*	.028	<.001	.092	.241
	Month 6	.099*	.023	<.001	.039	.159

Based on estimated marginal means
*. The mean difference is significant at the .05 level.
b. Adjustment for multiple comparisons: Bonferroni.

Table S6. Post hoc analysis of fasting blood glucose (FBG) among DMT2 patients

		Mean Difference	SE	P value.[b]	95% CI for Difference[b]	
					Lower Bound	Upper Bound
Baseline	Month 3	77.940*	1.330	<.001	74.417	81.464
	Month 6	93.044*	1.444	<.001	89.220	96.868
	Month 12	104.201*	1.499	<.001	100.231	108.172
Month 3	Baseline	-77.940*	1.330	<.001	-81.464	-74.417
	Month 6	15.104*	.538	<.001	13.678	16.529
	Month 12	26.261*	.632	<.001	24.587	27.935
Month 6	Baseline	-93.044*	1.444	<.001	-96.868	-89.220
	Month 3	-15.104*	.538	<.001	-16.529	-13.678
	Month 12	11.157*	.354	<.001	10.218	12.096
Month 12	Baseline	-104.201*	1.499	<.001	-108.172	-100.231
	Month 3	-26.261*	.632	<.001	-27.935	-24.587
	Month 6	-11.157*	.354	<.001	-12.096	-10.218

Based on estimated marginal means
*. The mean difference is significant at the .05 level.
b. Adjustment for multiple comparisons: Bonferroni.

Table S7. Post hoc analysis of posprandial blood glucose (PBG) among DMT2 patients

		Mean Difference	SE	P value.[b]	95% CI for Difference[b]	
					Lower Bound	Upper Bound
Baseline	Month 3	160.414*	1.832	<.001	155.561	165.268
	Month 6	172.721*	1.908	<.001	167.668	177.774
	Month 12	179.092*	1.923	<.001	173.999	184.184
Month 3	Baseline	-160.414*	1.832	<.001	-165.268	-155.561
	Month 6	12.307*	.641	<.001	10.608	14.006
	Month 12	18.677*	.779	<.001	16.614	20.741
Month 6	Baseline	-172.721*	1.908	<.001	-177.774	-167.668
	Month 3	-12.307*	.641	<.001	-14.006	-10.608
	Month 12	6.371*	.483	<.001	5.091	7.650
Month 12	Baseline	-179.092*	1.923	<.001	-184.184	-173.999
	Month 3	-18.677*	.779	<.001	-20.741	-16.614
	Month 6	-6.371*	.483	<.001	-7.650	-5.091

Based on estimated marginal means
*. The mean difference is significant at the .05 level.
b. Adjustment for multiple comparisons: Bonferroni.

Table S8. Post hoc analysis of HbA1C among DMT2 patients

		Mean Difference	SE	P value.[b]	95% CI for Difference[b]	
					Lower Bound	Upper Bound
Baseline	Month 3	2.053*	.034	<.001	1.963	2.144
	Month 6	2.951*	.045	<.001	2.833	3.070
	Month 12	3.292*	.045	<.001	3.174	3.410
Month 3	Baseline	-2.053*	.034	<.001	-2.144	-1.963
	Month 6	.898*	.021	<.001	.841	.955
	Month 12	1.238*	.023	<.001	1.178	1.299
Month 6	Baseline	-2.951*	.045	<.001	-3.070	-2.833
	Month 3	-.898*	.021	<.001	-.955	-.841
	Month 12	.340*	.012	<.001	.308	.373
Month 12	Baseline	-3.292*	.045	<.001	-3.410	-3.174
	Month 3	-1.238*	.023	<.001	-1.299	-1.178
	Month 6	-.340*	.012	<.001	-.373	-.308

Based on estimated marginal means
*. The mean difference is significant at the .05 level.
b. Adjustment for multiple comparisons: Bonferroni.
